# Use of Immersive Virtual Reality in Nursing Homes for People With Dementia: Feasibility Study to Assess Cognitive, Motor, and Emotional Responses

**DOI:** 10.2196/54724

**Published:** 2024-08-21

**Authors:** Alexander Prinz, Dan Buerger, Jelena Krafft, Matteo Bergmann, Alexander Woll, Bettina Barisch-Fritz, Kerstin Witte

**Affiliations:** 1 Department of Sports Engineering/Movement Science Otto-von-Guericke-University Magdeburg Magdeburg Germany; 2 Institute of Sports and Sports Science Karlsruhe Institute of Technology Karlsruhe Germany

**Keywords:** persons with dementia, virtual reality, VR, immersive virtual reality, iVR, head-mounted display, HMD, physical performance, physical activity, physical function, motor performance, Alzheimer’s disease, Alzheimer’s, Alzheimer’s treatment, Alzheimer’s care, Alzheimer’s symptom control, dementia, dementia therapy, dementia care, cognitive decline, cognitive impairment, cognitive impairments, neurocognition, neurology, neurologist, neurologists, nursing home, nursing homes, nursing facility, senior home, long-term care center, long-term care facility

## Abstract

**Background:**

Physical activity interventions for people with dementia have shown promising effects in improving cognition and physical function or slowing disease-related decline. Immersive virtual reality (iVR), using head-mounted displays, facilitates realistic experiences by blurring the boundaries between VR and the real world. The use of iVR for people with dementia offers the potential to increase active time and improve dementia therapy and care through exercise interventions. However, the feasibility of using VR use in people with dementia, considering changes in motor, cognitive, psychological, and physiological parameters, remains insufficiently investigated.

**Objective:**

This study aims to investigate the feasibility of using iVR in people with dementia or mild cognitive impairment in nursing homes. Specifically, we examined changes in motor performance (balance and mobility), cognitive performance (global cognition and executive functions), emotional responses, and fear of falling using iVR.

**Methods:**

Utilizing a pre-post design, this study recruited 35 participants with mild-to-moderate dementia, assessed by the Mini-Mental State Examination (MMSE). Participants underwent a single session involving iVR exposure, with pre- and postexposure assessments and a feedback form, to exclude negative effects on cognitive and motor functions, mood, anxiety levels, and balance performance. The use of iVR involved 4 scenes, with a total length of 8 minutes. These scenes depicted a park with short and rather passive impressions presented as a 360° video in a head-mounted display. Before and after using the iVR, cognitive parameters were assessed using the Trail-Making Test A (TMT-A), motor parameters were assessed using the FICSIT-4 (Frailty and Injuries: Cooperative Studies of Intervention Techniques-4) and Timed-Up-and-Go (TUG) tests, and psychological parameters were assessed using the Dementia Mood Picture Test, State-Trait Anxiety Inventory, and Short Falls Efficacy Scale-International (Short FES-I). The Emotion Rating Scale and the duration of use were recorded during use, and a feedback questionnaire was completed afterward in addition to the posttests. Paired *t* tests and Wilcoxon tests were used to examine pre-post differences.

**Results:**

Of the 35 initial participants, 33 completed the study, which corresponds to a dropout rate of 6%. All 33 participants, who had a mean of 83.71 (SD 5.01) years, had dementia. They showed no statistically significant difference in cognitive and motor performance before and after iVR use. Thus, no negative effects on cognitive and motor functions, mood, anxiety levels, and balance performance were observed. The emotion rating scale also showed that 72% (n=24) felt joy and fun during iVR use, 100% (n=33) showed no emotions such as fear, sadness, or anger, and 93% (n=31) were attentive during iVR use.

**Conclusions:**

The feasibility of using iVR for people with dementia can be rated positively. There were no changes in motor, cognitive, or emotional parameters that would increase the risk of falls or other negative emotional reactions during or after iVR use. Further studies are needed to investigate prolonged use in a more stimulating computer-generated environment and possible physical and cognitive tasks for people with dementia in nursing homes.

**Trial Registration:**

German Clinical Trials Register DRKS00030616; https://drks.de/search/de/trial/DRKS00030616

## Introduction

The number of people with dementia is expected to increase to over 152 million worldwide by 2050 [1], representing a high societal burden in terms of costs and care [2]. Dementia treatment is predominantly based on symptom reduction, with drug therapy often being associated with negative side effects [3], making treatment with nonpharmacological interventions important [4]. In particular, physical activity interventions have shown promising effects in improving cognition and physical function or slowing disease-related decline among people with dementia [5-9]. Additionally, quality of life and the ability to perform activities of daily living can also be positively affected by physical activity [7,10,11]. With the increasing use and dissemination of digital health applications in the past, there are new opportunities to complement or improve dementia care (ie, to respond to the individual needs of people with dementia) [12]. Virtual reality (VR) is a possibility that has great potential for treating cognitive impairment in the nursing home setting.

VR is based on computer simulations that allow individuals to enter an artificial environment. A distinction is made between nonimmersive, semi-immersive, and immersive VR technologies. Nonimmersive methods are based on desktop systems, with stereoscopic displays and head tracking, while semi-immersive methods are based on large single-screen or tabletop displays [13]. Immersive VR (iVR) technologies refer to the specification of the hardware (eg, the field of view, screen resolution, and refresh rate), as well as the degree of interaction and how isolated the user is from the outside world [14]. Clay et al [15] define iVR as “systems that encompass the user's field of view and where virtual motion replicates actual head or body motion.” For this purpose, head-mounted displays (HMDs) are usually used to create an even better 3D perspective to completely block out the real environment. Consequently, the immersive method leads to the most authentic experiences [16] and is therefore of particular interest.

VR has already been used in studies to improve cognitive function in both healthy individuals [17] and those with cognitive impairment [18] and to promote well-being in people with dementia [19]. The conclusion of a systematic review and meta-analysis review on the effects of VR-based task-oriented training on people with dementia or mild cognitive impairment (MCI) is that VR interventions are promising nonpharmacological approaches for improving cognitive and motor function [20]. Despite promising intervention effects [21-23], the feasibility of VR technologies in dementia therapy and care has not yet been sufficiently investigated [18]. Only a few studies with small sample sizes have examined the feasibility of VR in people with dementia [24]. In particular, direct changes in motor, cognitive, psychological, and physiological parameters, which are particularly important for the safety of people with dementia in everyday nursing home life, have scarcely been investigated following the use of VR technologies. In this context, it is important to take a closer look at cybersickness, which could increase the risk of falling after the use of VR technologies and is associated with injuries requiring hospitalization [25] and a decrease in activity, mobility, and quality of life [26]. Similarly, for an iVR intervention to be sustainable in nursing homes, there should be no deterioration in motor performance, confusion, and anxiety after iVR use. The feasibility of iVR in terms of safe use in people with dementia regarding influencing motor, cognitive, or emotional factors is currently inconclusive [27,28].

Therefore, the primary aim of this study is to investigate the feasibility of using iVR in people with dementia or MCI in nursing homes from a safety perspective. The focus of this study is to investigate any adverse effects of iVR on motor and cognitive function and general well-being. In particular, this study examines motor performance (including balance and mobility), cognitive performance (such as global cognition and executive function), emotional responses, and fear of falling before and after the single iVR use. This study can be seen as a preliminary step in assessing the suitability of iVR for physical training in people with dementia in nursing homes.

## Methods

### Ethical Considerations

The research protocol followed the Declaration of Helsinki and ethical approval was granted by the Ethics Committee of the Otto-von-Guericke University Magdeburg (number 131/22) and the Karlsruhe Institute of Technology. The study was registered in the German Clinical Trials Register (DRKS00030616). The participants and their legal guardians provided written informed consent after being briefed on the study's details. Data were collected from December 2022 to July 2023. Participants were not offered any incentives/compensation for participation.

### Study Design and Sample

**Figure 1:**
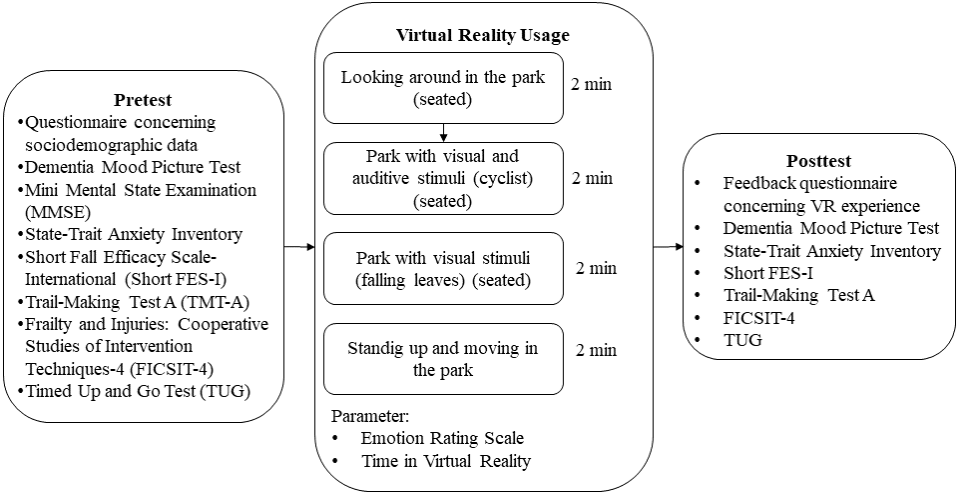
Design of the study.

The study was designed as a pre-post study to examine possible side effects of iVR by comparing motor and cognitive functions and emotional responses before and after iVR use (see Figure 1). All measurements were carried out in a single session lasting approximately 45 minutes. In each of the 2 cities, Magdeburg and Karlsruhe, 3 facilities were contacted to identify potential participants with the help of nursing home staff. The following inclusion criteria were applied: age over 70 years, MCI or mild to moderate dementia based on the Mini-Mental State Examination (MMSE [29]), no untreated hearing or visual impairment, and self-sufficiency in standing and walking unaided or with a rollator. Exclusion criteria included hypertension, severe cardiovascular disease, and significant motor impairment. To check the inclusion criteria, a questionnaire on the sociodemographic data was completed together with the participants’ legal guardians before the start of the study. Initially, 35 participants were included in the study, 23 (66%) in Magdeburg and 12 (34%) in Karlsruhe. Prior to the baseline assessments, the participants and their legal guardians were informed about the aims of the study, and written informed consent was obtained from the participants' legal guardians.

### Test Battery

To test the feasibility of iVR in people with dementia and its impact on motor performance, cognitive performance, and emotional response, the participants underwent several tests before and after the iVR application (Figure 1). The tests assessed the participants' mood using the Dementia Mood Picture Test [30], state and trait anxiety using the State-Trait Anxiety Inventory [31], fear of falling using the Short Falls Efficacy Scale-International (Short FES-I) [32], attention using the Trail-Making Test A (TMT-A) [33], balance performance using the FICSIT-4 (Frailty and Injuries: Cooperative Studies of Intervention Techniques-4) scale [34], and mobility using the Timed-Up-and-Go test (TUG) [35]. During the iVR use, the Emotion Rating Scale and another questionnaire adapted from Appel et al [27] (Multimedia Appendix 1) were applied. Additionally, after removing the HMD, participants completed a feedback questionnaire about their experience and general interest in VR.

In the Dementia Mood Picture Test, the participants are presented with 6 emoticons expressing different moods (negative mood, positive mood, cheerfulness, concern, sadness, and anger). Participants are asked to choose the emoticon that best describes their current mood. Cheerful is considered the best, followed by a positive mood. In the State-Trait Anxiety Inventory, participants are asked to rate their general and current emotional state. A score is calculated based on the answers given, with higher scores being associated with higher levels of anxiety (state and trait max. 80 points [31]). The Short FES-I asks participants about their fear of falling in various situations. The score is also calculated based on the answers given, and again, with higher scores associated with a greater fear of falling (maximum 28 points [32]). To complete the TMT-A, the participants enter numbers 1 to 25 (jumbled together on a paper) in ascending order. The time taken and the number of errors made are recorded, with a shorter time and fewer errors associated with better attention. The FICSIT-4 tests 4 successive levels of static balance (bilateral stance, semitandem stance, tandem stance, and unilateral stance), each performed with eyes open and closed. Points are awarded according to the duration of each level (maximum 10 seconds). These points are summed up to give a total score (maximum 28 points), with higher scores associated with better static balance performance [34]. For the TUG test, participants are asked to stand up from a chair, walk 3 meters, turn 180°, walk back to the chair, and sit down again. The time taken to complete this task is measured, with a shorter time associated with a lower risk of falling [35].

The Emotion Rating Scale was scored by a test instructor during iVR use by observing participants' facial expressions. The emotional expressions (happiness, anger, fear, sadness, and general attention) and their duration (never, less than 16 seconds, 16 to 59 seconds, 1 to 5 minutes, and over 5 minutes) were documented.

The feedback questionnaire (Multimedia Appendix 1), which was administered after the iVR use, consisted of 17 questions (eg, “Did you like the VR?”), which were scored from one to five points, with more points indicating a higher level of agreement. The answers to negative questions (eg, “Did you feel nauseous while watching the VR?”) were inverted. The questions were divided into 3 dimensions: “Response to VR” (questions 2, 6, 7, 10, 11, 15, 16), “Feedback to VR” (questions 1, 3, 4, 5, 8, 9), and “Comfort” (questions 12, 13, 14, and 17). The points were later summed up to dimension scores and a total score (maximum 85 points).

### Virtual Reality Setup

**Figure 2:**
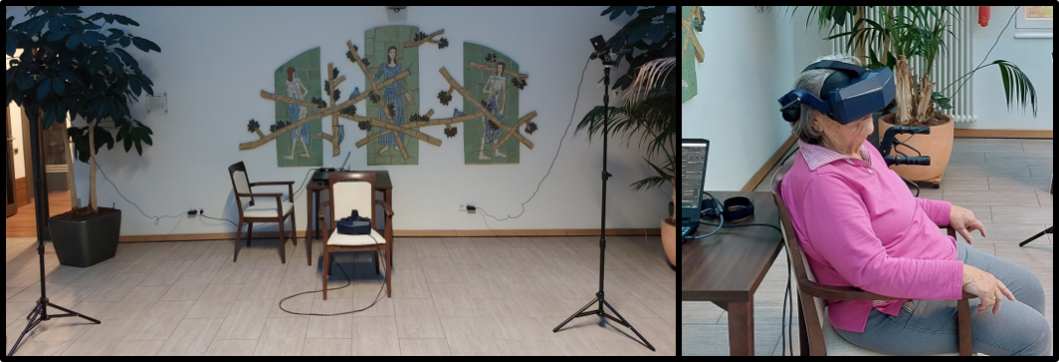
Virtual reality setup. Chair between 2 SteamVR Base Stations 2.0 with Pimax Vision 5k super (top). Participant with head-mounted display (HMD) and virtual park environment (bottom).

During the iVR use, the participants sat on a chair between 2 SteamVR Base Stations (version 2.0; HTC Corp), which tracked the position of the HMD (Figure 2). The Pimax Vision 5k super (Pimax Inc) (170° horizontal FOV, 2560 x 1440 pixels per eye, 90 Hz refresh rate) was wired to a computer via cable and presented the virtual environments, which consisted of different scenes provided as 360° videos recorded in the Geschwister-Scholl-Park in Magdeburg, using the GoPro Hero 8 (GoPro Inc) (Figure 2). These videos were projected onto the inner surface of a virtual sphere (radius of 50 m) modeled with Blender (version 3.2; Blender Foundation). The virtual environments were then constructed using Unity (Unity Technologies). Self-written C# scripts were used to enable dynamic scene control within iVR, by allowing seamless scene transitions and pauses with simple button presses on a standard keyboard used by the test instructors. SteamVR (version 1.25.1; Valve Corp) was used to transfer the scenes from Unity to the HMD.

### Procedure

**Figure 3:**
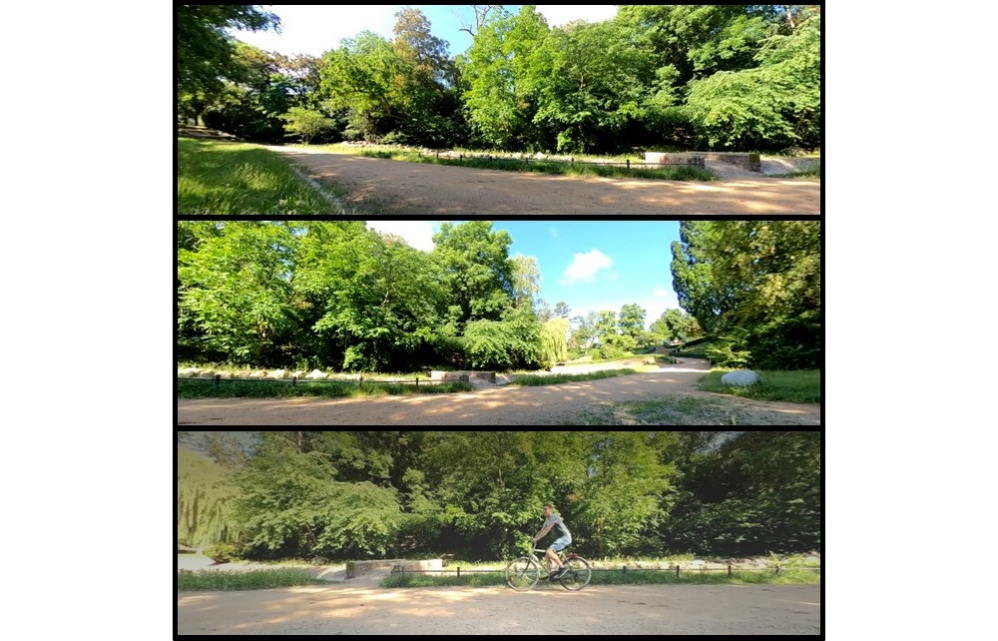
Immersive virtual reality (iVR) park environment (top, mid) with cyclist (bottom).

After the pretest, the iVR use began, consisting of 4 scenes, each showing the same specific section of a park presented as a 360° video for 2 minutes. First, the HMD was demonstrated to the participants and placed on their heads. Once they were comfortable, the first scene began, showing the park without any additional elements (see Figure 3). During the first scene, the participants were asked to look around and describe the environment. The second scene showed a cyclist riding through the same section of the park, signaling their presence by ringing a bell. In the third scene, the participants saw leaves falling to the ground, controlled by the instructor pressing the space bar on the computer keyboard. The final scene was identical to the first, but the participants were asked to stand up and walk a few steps if they wished and if the instructors and nursing staff felt it was feasible. While standing, the participants were always secured by at least 2 instructors. The scenes were presented directly, one after the other, without a break. After the last scene, the HMD was removed from the participants' heads, and the feedback questionnaire was administered. The posttest was then completed to detect deviations from the baseline measurement.

### Statistical Analysis

Statistical data analysis was performed using SPSS (version 28.0; IBM Corp). The pre- and posttests (State-Trait Anxiety Inventory, Short FES-I, TMT-A, FICSIT-4, and TUG) were compared using paired *t* tests. If the prerequisites for a *t* test were unmet, the Wilcoxon test was used instead. The significance level was set at α=.05. To avoid the cumulation of alpha errors, the significance level was adjusted using the Bonferroni-Holm correction. Cohen classification was used to interpret the effect sizes (*d*=0.2/*r*= 0.1, small; *d*=0.5/*r*=0.3, moderate; *d*=0.8/*r*= 0.5, large). The Dementia Mood Picture Test, MMSE, Emotion Rating Scale, and feedback questionnaire were analyzed descriptively.

## Results

### Sample characteristics

Two participants dropped out during the study, corresponding to a dropout rate of 6%. The first participant signaled disinterest at the beginning of the measurement day and dropped out immediately, while the other participant was bothered by the HMD and wanted to take it off. Thus, 33 (94%) of the 35 people with dementia (mean age 83.71, SD 5.01 years) were analyzed in the study. Table 1 shows the sample characteristics.

Care was taken to ensure that participants with dementia had no untreated visual or hearing impairments. The HMD could also be used with visual and/or hearing aids.

**Table 1:** Sample characteristics.

Characteristics		Sample (N=33)
Age (years), mean (SD)		83.71 (5.01)
MMSE^a^ score, mean (SD)		21.2 (4.2)
**Sex, n (%)**		
	Male	7 (21.2)
	Female	26 (78.8)
Experience with VR^b^; n (%)		6 (18.2)
**Degree of dementia n (%)**		
	No dementia	1 (3)
	Mild dementia	21 (63.6)
	Moderate dementia	11 (33.3)

^a^MMSE: Mini-Mental State Examination.

^b^VR: virtual reality.

### Outcome Parameters

#### Feasibility

**Figure 4:**
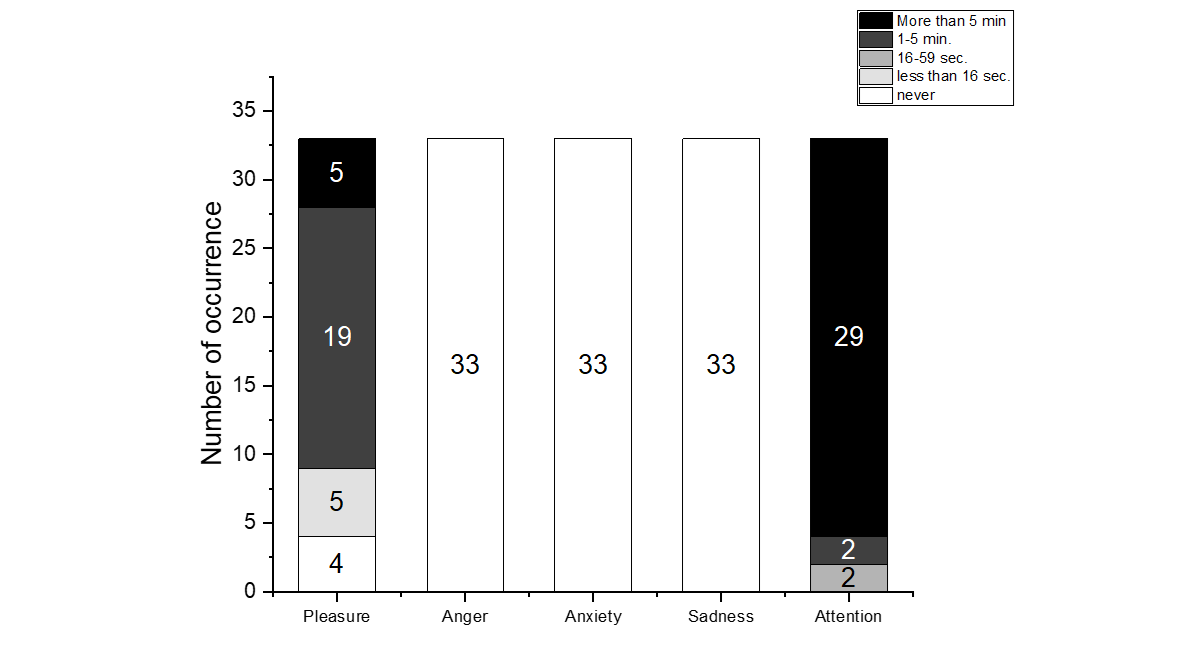
Results of the Emotion Rating Scale.

Observations of the participants during the iVR use revealed that 72% (n=24) felt pleasure and fun, as measured by the Emotion Rating Scale. Additionally, all 33 (100%) participants showed no fear, sadness, or anger during the iVR use, and 31 (93%) out of 33 patients were attentive and able to respond to everything in the environment and thus interact with the iVR (Figure 4).

**Figure 5:**
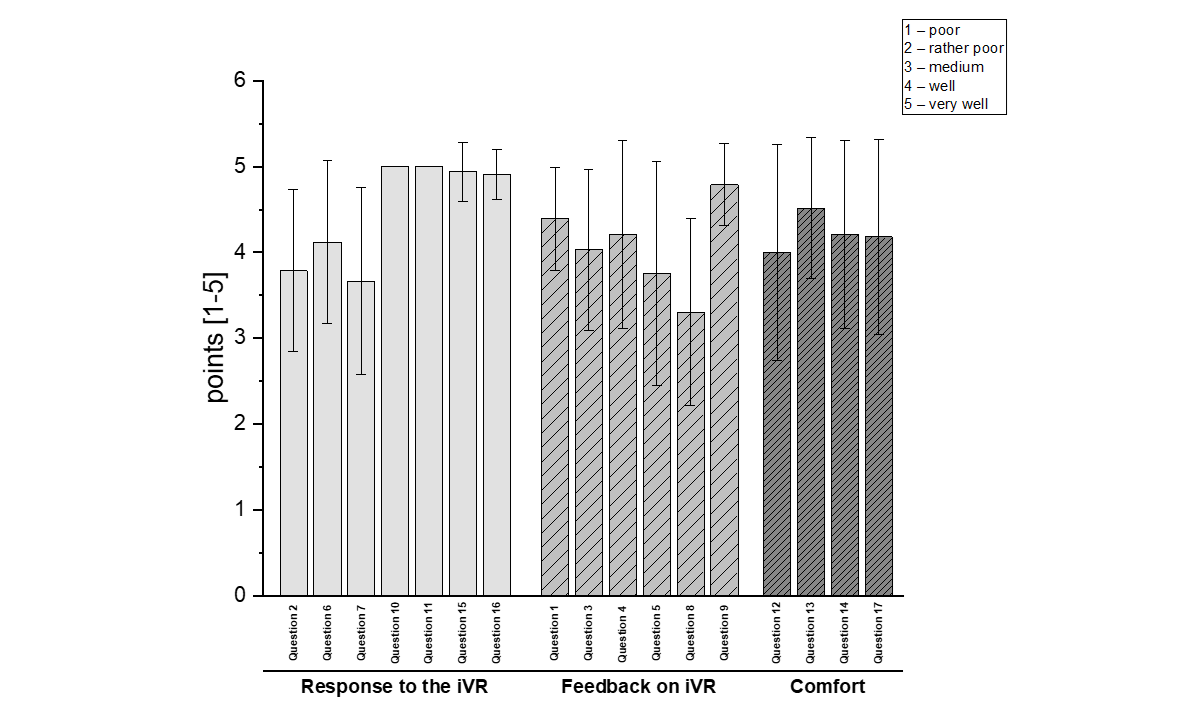
Results of the protocol response after immersive virtual reality (iVR) use.

The feedback form according to Appel et al [27] showed that the participants rated the iVR as positive on average. The total score was 73.21 (SD 7.1) out of a possible 85 points. This is also reflected in the individual dimension of the questionnaire, as outlined in the Methods section. The participants responded very positively to the iVR use. On average, the participants rated this dimension 31.2 out of 35 points. The participants also gave positive feedback on the iVR. by rating this dimension with 24.9 out of 30 points. The final dimension, comfort, was rated positively with 17 out of 20 points (see Figure 5). The questions of the dimensions can be found in Multimedia Appendix 1. Additionally, the actual time spent in iVR was recorded showing an average of 389 seconds (6.5 seconds minimum; the maximum was 480 seconds or 8 minutes). The first 3 scenes (Figure 1) were completed without issue; some participants did not want to try scene 4 (standing up).

#### Motor and Cognitive Performance and Mental State

The results show no significant changes in cognitive and motor performance and mental state after iVR (Table 2) (TMT-A: t_26_=1.244, correct *P*>.99; state anxiety: t_32_=1.653, correct *P*=.65; trait anxiety: t_32_=1.754, correct *P*=.53; FICSIT-4: t_29_=-2.531, correct *P*=.16; TUG: t_28_=-0.036, correct *P*>.99; short FES-I: *z*=-1.461, correct *P*=.68). However, almost all parameters showed slight but not statistically significant improvement after iVR use. On the TMT-A, people with dementia improved by a mean of 9.5 seconds. State anxiety and trait anxiety improved by 2.65 and 3.39 points, respectively. The FICSIT-4 showed an increase of 1.44 points from pre- to posttest. The results of the TUG and the short FES-I did not change.

The Dementia Mood Picture Test also reflects a slight improvement: 27 (82%) out of 33 participants were in a good mood before iVR, and 6 (18%) were cheerful. After iVR, the distribution of the 2 moods was balanced so that 16 (49%) out of 33 participants were in a good mood and 17 (51%) were in a cheerful mood.

**Table 2:** Results of the pre- and posttest.

Outcomes		Pretest, mean (SD) (95% CI)	posttest, mean (SD) (95% CI)	*P* value (Bonferroni-Holm)	Effect size (*d*)
**Parametric tests (*t* test) **					
	TMT-A^a^ (n=27)	118.43 (70.10) (90.67-146.20)	108.91 (80.60) (77.01-140.8)	.225 (>.999)	0.24
	State anxiety (points) (n=33)	33.94 (8.36) (29.42-36.46)	31.29 (10.25) (26.31-34.28)	.108 (.648)	0.28
	Trait anxiety (points) (n=33)	33.45 (8.69) (28.87-36.07)	30.06 (10.51) (25.14-33.21)	.089 (.534)	0.30
	FICSIT-4^b^ (points); n=30	15.63 (5) (13.76-17.51)	17.07 (5.10) (15.16-18.98)	.026 (.156)	0.43
	TUG^c^ (n=29)	15.53 (5.60) (13.41-17.65)	15.56 (5.50) (13.47-17.65)	.976 (>.999)	0.007
**Nonparametric tests (Wilcoxon test)**					
	Short FES-I^d^ (points) (n=33)	7.50^a^ (7.00-11.25)	7.00^a^ (7.00-10.00)	.114 (.684)	0.25^e^

^a^TMT-A: Trail-Making Test A.

^b^FICSIT-4: Frailty and Injuries: Cooperative Studies of Intervention Techniques-4.

^c^TUG: Timed-Up-and-Go.

^d^FES-I: Falls Efficacy Scale-International.

^e^ Median and 25th/75th percentile, Correlation coefficient *r*.

## Discussion

### Principal Findings and Comparison to Prior Work

This study demonstrates the feasibility of using iVR in people with dementia without significant changes or effects on emotional, cognitive, or physical outcomes. Thus, no negative side effects were observed following iVR use. Out of 35 participants, only 2 dropped out during the study, and only 1 of these was due to the participant feeling disturbed by the HMD. Thus, the dropout rate for iVR use was low, which is comparable to Appel et al [27], who observed iVR use in people with cognitive and/or physical impairments and Thapa et al [22] who analyzed iVR use in people with MCI. Liao et al [23] reported a higher dropout rate (8 out of 42) in people with MCI due to low motivation during the iVR use, but this was a 12-week intervention. Other studies using VR in people with dementia did not report dropout rates, so no further comparisons are available [36,37].

In general, it might have been expected that people with dementia would have challenges participating in the iVR [38]. On the one hand, there is a lack of haptic feedback, and on the other, there is acoustic information from the real world (eg, instructors speaking and birds singing in the iVR at the same time). The feasibility of using iVR can be positively rated by the average time participants used the iVR, which was 6.5 minutes (out of a maximum of 8 minutes). Additionally, the reasons for discontinuing iVR use, which were more due to the low-stimulus environment rather than excessive demands or overstimulation, also indicate good feasibility. The first 3 scenes of iVR use could be followed by all participants without any issues.

Most of the participants (n=31, 93%) were attentive during the iVR experience, which has not been reported previously. As far as we know, Mendez et al [36] only found that the participants' talkativeness increased while wearing an outdated HMD that did not allow full immersion. Pleasant feelings such as joy or fun were observed in the majority of participants, particularly in 5 (15%) participants over a longer period (>5 min). This is a positive observation, and it echoes the study by Appel et al [27], where VR exposure was generally considered to be enjoyable. In addition to the positive emotional reactions, the question arises as to whether confusion, which occurs disproportionately often in people with dementia [39], is related to the use of VR. Confusion or challenging behavior may be associated with extreme or extremely negative emotions [40], which we sought to observe during the iVR use. In our study, no participant showed anxiety, sadness, or anger during iVR use. Thus, our findings agree with those of Appel et al [27] and Mendez et al [36] regarding negative emotions.

The emotional observations are particularly important for future long-term use in an iVR intervention, as a sustainable intervention in nursing homes should only be implemented if additional benefits in the real world are observed. The joy and attention experienced, combined with the absence of fear, anger, and sadness, suggest a positive impact on the quality of life. Regarding challenging behavior among people with dementia in nursing homes and home care [41], future studies could also investigate whether positive outcomes can be achieved through iVR use.

Since people with dementia are known to face challenges in correctly identifying and associating negative emotions [42], the questions about the iVR use were particularly relevant and helpful in correctly classifying the observations. Our results show that participants generally rated the iVR use as positive, as assessed by the feedback questionnaire based on Appel et al [27]. The responses to the questions are summarized under the 3 dimensions of response, feedback, and comfort.

The general response to the iVR use was assessed by questionnaire items such as, “This helped you to relax and free yourself from unwanted feelings or thoughts,” “You felt like you were panicking when you saw this,” or “Did you get dizzy while watching VR?.” All questions about dizziness or nausea, as well as confusion or disorientation, were answered in the negative, so no negative effects are to be expected. Similar results were also reported by Appel et al [27], who found that 92% of respondents did not experience nausea while using VR and that other side effects such as dizziness, disorientation, or confusion were not detected. The general response to the use of iVR can therefore be considered positive, which was also shown in the feedback subdimension. Questionnaire items were asked regarding this realm, such as, “The virtual world appeared to be very real to you,” “This was fascinating to observe,” or “You want to spend more time viewing this environment.”

Questions about the comfort of the HMD included whether participants were able to get used to the HMD, whether they found the HMD heavy, and whether they were able to move their heads well. High scores were found for these items, so the comfort of the HMD can be rated as very good. This was also reported by Appel et al [27], where 88% of the participants negated the question, “Did the VR HMD feel too heavy?” In general, however, this assessment must be qualified in the sense that the participants in our study only used the HMD for a relatively short period of time and did not perform any guided movements with it.

There were no statistically significant changes in motor and cognitive performance or mental state after the single use of iVR. Side effects from iVR use can therefore be ruled out. This is a particularly positive finding for people with dementia, as it could be expected that negative effects such as dizziness or nausea may occur after just a few minutes of using iVR [38]. The nonsignificant improvements in almost all tests and questionnaires after iVR use suggest a learning effect due to the short time interval between measurements. Assumptions that the improvements are also due to an improvement in mood, as has been found in people with depression [43], are speculative and need to be investigated in further studies. To assess the changes, it is important that physical and cognitive performance does not decline significantly after the use of iVR, as this would lead to an increased risk of falls and thus more intensive care.

State anxiety and trait anxiety also improved and may also be related to positive emotions. The more positive emotions were reflected in a change in the Dementia Mood Picture Test, which showed better mood scores after iVR use, along with cheerful feelings, which may be related to a more positive mood. While the results for mobility and fear of falling remained the same, there were changes in balance after iVR use. This has not been previously reported and cannot be explained presently. The mean differences in the FICSIT-4 test meet the threshold of the minimal clinically important difference [44], but the results are not statistically significant and can therefore only be interpreted as trends. This may indicate a possible effect of longer-term interventions, which needs to be verified in future studies. In addition to the findings on balance, mobility, and fear of falling, we observed that some participants did not want to try to stand up with the HMD iVR system. This suggests that some participants may have felt unsafe while using iVR, which needs to be considered for future iVR interventions, especially those involving increased physical activation.

### Strengths and Limitations

The strength of this study is the use of iVR for people with dementia under real conditions in nursing homes. To date, there have been few studies of this nature, and we were able to show that the use of iVR in nursing homes is feasible and is not expected to have a negative impact on people with dementia.

As with any study, there are several limitations. The first is the rather low-stimulation VR environment, where there was no direct activation in the form of physical activity. The rationale was to investigate the general feasibility and gain experiences with iVR for people with dementia to assess and possibly minimize the risks of such VR uses. This was particularly necessary for this target group to gain a first impression and can therefore be seen as a preliminary step in assessing the suitability of iVR for physical activity in people with dementia in nursing homes. We aimed to avoid situations that could lead to cognitive disorientation, emotional reactions, or physical responses such as dizziness. The second limitation is the relatively small sample size, which, although considered appropriate for the study design and objectives, does not allow for generalization. Additionally, the sample had a rather mild degree of dementia, which limits the generalizability to the whole population of people with dementia. The third limitation is the single use of iVR in a predominantly seated position. The single use of iVR and the predominantly seated position influenced the interaction and incentive to be physically active, at the same time legitimizing and strengthening the use of iVR to promote physical activity. Therefore, the duration of the intervention, the sample included, and a critical review of the cost-benefit ratio need to be included in future studies.

### Future Directions

Future studies should build on these findings by including more stimulating environments that encourage more physical activity. Such environments could potentially increase engagement and movement to exercise, overcoming the limitations of this study’s low-stimulation VR environment. To this end, the feasibility of computer-generated virtual environments for people with dementia should be examined, as they provide a higher degree of individualization and interaction compared to 360° videos. Additionally, longer-term iVR interventions with multiple sessions should be investigated to assess lasting effects on motor and cognitive performance. Future research should also investigate the potential of iVR to positively impact the quality of life of people with dementia by examining its effects on mood, emotional well-being, and general engagement. Finally, the economic feasibility of implementing iVR interventions needs to be considered. Future studies should include cost-benefit analyses to determine the overall value and feasibility of iVR as a regular intervention in care settings. By addressing these areas, future research can further elucidate the potential of iVR to improve the well-being of people with dementia and contribute to the development of effective and scalable interventions.

### Conclusion

The changes observed in our study due to the use of iVR do not indicate that the use of iVR increases the risk of falling or causes adverse effects such as dizziness or nausea. Therefore, we can conclude that iVR use can be applied in nursing homes for people with dementia or MCI and that no debilitating emotional, cognitive, or physical changes are to be expected afterward. However, care must be taken during iVR use to ensure that there are no events that increase the risk of falling. In this study, which involved a single use of iVR in a predominantly seated position and a relatively low stimulus iVR environment, there were no complications. Further studies are needed to investigate prolonged use with a more stimulating environment and possible physical and cognitive tasks in people with dementia.

## Appendix

Multimedia Appendix 1 Assessment battery.

## Abbreviations

FICSIT-4: Frailty and Injuries: Cooperative Studies of Intervention Techniques-4
HMD: head-mounted display
iVR: immersive virtual reality
MCI: mild cognitive impairment
MMSE: Mini-Mental State Examination
Short FES-I: Short Falls Efficacy Scale-International
TMT-A: Trail-Making Test A
TUG: Timed-Up-and-Go Test
VR: virtual reality

## Data Availability

The datasets used and/or analyzed during the current study are available from the corresponding author upon reasonable request.

## References

[1] GBD 2019 Dementia Forecasting Collaborators (2022). Estimation of the global prevalence of dementia in 2019 and forecasted prevalence in 2050: an analysis for the Global Burden of Disease Study 2019. Lancet Public Health.

[2] Global status report on the public health response to dementia. World Health Organization.

[3] Jönsson L, Tate A, Frisell O, Wimo A (2023). The costs of dementia in Europe: an updated review and meta-analysis. Pharmacoeconomics.

[4] Bessey LJ, Walaszek A (2019). Management of behavioral and psychological symptoms of dementia. Curr Psychiatry Rep.

[5] Farina N, Rusted J, Tabet N (2013). The effect of exercise interventions on cognitive outcome in Alzheimer's disease: a systematic review. Int Psychogeriatr.

[6] Sofi F, Valecchi D, Bacci D, Abbate R, Gensini GF, Casini A, Macchi C (2011). Physical activity and risk of cognitive decline: a meta-analysis of prospective studies. J Intern Med.

[7] Zhou S, Chen S, Liu X, Zhang Y, Zhao M, Li W (2022). Physical activity improves cognition and activities of daily living in adults with Alzheimer's disease: a systematic review and meta-analysis of randomized controlled trials. Int J Environ Res Public Health.

[8] Deslandes A, Moraes H, Ferreira C, Veiga H, Silveira H, Mouta R, Pompeu FA, Coutinho ESF, Laks J (2009). Exercise and mental health: many reasons to move. Neuropsychobiology.

[9] Begde A, Jain M, Hogervorst E, Wilcockson T (2022). Does physical exercise improve the capacity for independent living in people with dementia or mild cognitive impairment: an overview of systematic reviews and meta-analyses. Aging Ment Health.

[10] Logsdon R, McCurry SM, Teri L (2007). Evidence-based interventions to improve quality of life for individuals with dementia. Alzheimer's Care Today.

[11] Liang Y, Su Q, Sheng Z, Weng Q, Niu Y, Zhou H, Liu C (2022). Effectiveness of physical activity interventions on cognition, neuropsychiatric symptoms, and quality of life of Alzheimer’s disease: an update of a systematic review and meta-analysis. Front Aging Neurosci.

[12] Neves A, Lygidakis C, Hoedebecke K, De PL, Pilotto A (2022). Digital Health in an Ageing World.

[13] Dörner R, Broll W, Grimm P, Jung B (2019). Virtual und Augmented Reality (VR/AR): Grundlagen und Methoden der Virtuellen und Augmentierten Realität.

[14] Dargar S, Kennedy R, Lai W, Arikatla V, De S (2015). Towards immersive virtual reality (iVR): a route to surgical expertise. J Comput Surg.

[15] Clay F, Howett D, FitzGerald J, Fletcher P, Chan D, Price A (2020). Use of Immersive Virtual Reality in the Assessment and Treatment of Alzheimer's Disease: A Systematic Review. J Alzheimers Dis.

[16] Slater M, Sanchez-Vives MV (2016). Enhancing our lives with immersive virtual reality. Front Robot AI.

[17] Skurla MD, Rahman AT, Salcone S, Mathias L, Shah B, Forester BP, Vahia IV (2021). Virtual reality and mental health in older adults: a systematic review. Int Psychogeriatr.

[18] Kim O, Pang Y, Kim J (2019). The effectiveness of virtual reality for people with mild cognitive impairment or dementia: a meta-analysis. BMC Psychiatry.

[19] Appel L, Ali S, Narag T, Mozeson K, Pasat Z, Orchanian-Cheff A, Campos JL (2021). Virtual reality to promote wellbeing in persons with dementia: A scoping review. J Rehabil Assist Technol Eng.

[20] Zhu S, Sui Y, Shen Y, Zhu Y, Ali N, Guo C, Wang T (2021). Effects of virtual reality intervention on cognition and motor function in older adults with mild cognitive impairment or dementia: a systematic review and meta-analysis. Front Aging Neurosci.

[21] Wiebe A, Kannen K, Selaskowski B, Mehren A, Thöne AK, Pramme L, Blumenthal N, Li M, Asché L, Jonas S, Bey K, Schulze M, Steffens M, Pensel MC, Guth M, Rohlfsen F, Ekhlas M, Lügering H, Fileccia H, Pakos J, Lux S, Philipsen A, Braun N (2022). Virtual reality in the diagnostic and therapy for mental disorders: A systematic review. Clin Psychol Rev.

[22] Thapa N, Park HJ, Yang J, Son H, Jang M, Lee J, Kang SW, Park KW, Park H (2020). The effect of a virtual reality-based intervention program on cognition in older adults with mild cognitive impairment: a randomized control trial. J Clin Med.

[23] Liao Y, Chen I, Lin Y, Chen Y, Hsu W (2019). Effects of virtual reality-based physical and cognitive training on executive function and dual-task gait performance in older adults with mild cognitive impairment: a randomized control trial. Front Aging Neurosci.

[24] Strong J (2020). Immersive virtual reality and persons with dementia: a literature review. J Gerontol Soc Work.

[25] Na'emani F, Esmaiil Zali M, Sohrabi Z, Fayaz-Bakhsh A (2019). Prevalence of risk factors for falls among the elderly receiving care at home. Salmand.

[26] Manckoundia P, Mourey F, Pérennou D, Pfitzenmeyer P (2008). Backward disequilibrium in elderly subjects. Clin Interv Aging.

[27] Appel L, Appel E, Bogler O, Wiseman M, Cohen L, Ein N, Abrams HB, Campos JL (2019). Older adults with cognitive and/or physical impairments can benefit from immersive virtual reality experiences: a feasibility study. Front Med (Lausanne).

[28] Canning CG, Allen NE, Nackaerts E, Paul SS, Nieuwboer A, Gilat M (2020). Virtual reality in research and rehabilitation of gait and balance in Parkinson disease. Nat Rev Neurol.

[29] Folstein MF, Folstein SE, McHugh PR (1975). "Mini-mental state". A practical method for grading the cognitive state of patients for the clinician. J Psychiatr Res.

[30] Tappen RM, Barry C (1995). Assessment of affect in advanced Alzheimer's disease: the Dementia Mood Picture Test. J Gerontol Nurs.

[31] Spielberger C, Gorsuch R, Lushene R Manual for the State-Trait Anxiety Inventory. Google Scholar.

[32] Kempen GIJM, Yardley L, van Haastregt JCM, Zijlstra GAR, Beyer N, Hauer K, Todd C (2008). The Short FES-I: a shortened version of the falls efficacy scale-international to assess fear of falling. Age Ageing.

[33] Reitan R (1955). The relation of the trail making test to organic brain damage. J Consult Psychol.

[34] Rossiter-Fornoff JE, Wolf SL, Wolfson LI, Buchner DM (1995). A cross-sectional validation study of the FICSIT common data base static balance measures. Frailty and Injuries: Cooperative Studies of Intervention Techniques. J Gerontol A Biol Sci Med Sci.

[35] Podsiadlo D, Richardson S (1991). J Am Geriatr Soc.

[36] Mendez MF, Joshi A, Jimenez E (2015). Virtual reality for the assessment of frontotemporal dementia, a feasibility study. Disabil Rehabil Assist Technol.

[37] Fernandez Montenegro JM, Argyriou V (2017). Cognitive evaluation for the diagnosis of Alzheimer's disease based on Turing test and virtual environments. Physiol Behav.

[38] Huygelier H, Schraepen B, van Ee R, Vanden Abeele V, Gillebert CR (2019). Acceptance of immersive head-mounted virtual reality in older adults. Sci Rep.

[39] Abraha I, Rimland JM, Trotta FM, Dell'Aquila G, Cruz-Jentoft A, Petrovic M, Gudmundsson A, Soiza R, O'Mahony D, Guaita A, Cherubini A (2017). Systematic review of systematic reviews of non-pharmacological interventions to treat behavioural disturbances in older patients with dementia. The SENATOR-OnTop series. BMJ Open.

[40] Ooi CH, Yoon PS, How CH, Poon NY (2018). Managing challenging behaviours in dementia. Singapore Med J.

[41] Feast A, Orrell M, Charlesworth G, Melunsky N, Poland F, Moniz-Cook E (2016). Behavioural and psychological symptoms in dementia and the challenges for family carers: systematic review. Br J Psychiatry.

[42] Hwang S, Hwang J, Jeong H (2022). Study on associating emotions in verbal reactions to facial expressions in dementia. Healthcare (Basel).

[43] Kraft B, Bø R, Jonassen R, Heeren A, Ulset VS, Stiles TC, Landrø NI (2023). The association between depression symptoms and reduced executive functioning is primarily linked by fatigue. Psychiatry Research Communications.

[44] Blankevoort CG, van Heuvelen MJG, Scherder EJA (2013). Reliability of six physical performance tests in older people with dementia. Phys Ther.

