# Supplementary material for: Use of Immersive Virtual Reality in Nursing Homes for People With Dementia: Feasibility Study to Assess Cognitive, Motor, and Emotional Responses
Source: JMIR XR Spat Comput. 2024 Aug 21;1:e54724. doi: 10.2196/54724 (PMC13179109; doi:10.2196/54724)
Supplement: Multimedia Appendix 1 [file xr_v1i1e54724_app1.docx]

**Supplement**

| **Dimension responses to the VR** | |
| --- | --- |
| Question 2 | This helped you to relax and free yourself from unwanted feelings or thoughts. |
| Question 6 | Watching this was boring. |
| Question 7 | You had a lot of fun watching this. |
| Question 10 | You felt like you were panicking when you saw this. |
| Question 11 | You felt confused or disoriented |
| Question 15 | Did you feel nauseous while watching the VR? |
| Question 16 | Did you get dizzy while watching the VR? |
| **Dimension feedback on VR** | |
| Question 1 | The virtual world appeared to be very real to you. |
| Question 3 | Your attention was focused on many interesting things. |
| Question 4 | You would like to see more places like this. |
| Question 5 | You want to spend more time viewing this environment. |
| Question 8 | This was fascinating to observe. |
| Question 9 | There was too much going on. |
| **Dimension comfort** | |
| Question 12 | Did you find it easy to get used to the VR headset? |
| Question 13 | Did you find the VR headset too heavy? |
| Question 14 | Did the VR headset affect you? |
| Question 17 | Could you easily move your head up and down and to the side to see more of your environment? |

Table 1. Cognitive test procedures

| **Cognition** | |
| --- | --- |
| **Mini-Mental State Examination ( Folstein, Folstein & McHugh, 1975)** | |
| Cognitive area | Declaration |
| General cognitive function level (Screening) | - Answering questions to determine the cognitive abilities of older people  o Orientation  o Absorption capacity  o Attention and calculation  o Memory  o Language  o constructive practice  - maximum 30 points |
| **Quality criteria:**    **-** Reliability: Interrater reliability: ICC = .83 - .95  - Test-restest reliability: r_tt_=.84  - Validity:  o Criterion validity: Relatively good differentiation between healthy and demented individuals, but less sensitive in the MCI domain.  o Construct validity: moderate correlation with other screening and rating methods (r=.43 - .70 with DemTect, r= .75 - .78 with SKT, r= .55 - .76 with ADAS-Cog) and Everyday competence (r= .28 with B-ADL) | |
| **Trail-Making- Test A** | |
| Cognitive area | Declaration |
| Psychomotor speed,  Executive functions | - number linking as fast as possible (from 1-25)  - - max. 180 s |
| **Quality criteria:** | |

Table 2 State-Trait Anxiety Inventory

| **State-Trait Anxiety Inventory ( Laux, Glanzmann, Schaffner & Spielberger, 1981)** | |
| --- | --- |
| Area | Declaration |
| Fear as a state and fear as a property | - two scales of the STAI with 20 items each are used to capture anxiety as a state (state anxiety) and anxiety as a trait (trait anxiety)  - 4s Likert scale  - Sum values from 20 - 80, where higher values stronger expressions of the respective anxiety trait show |
| **Quality criteria:**    - Internal consistency for both scales α = .90  - Test-restest reliability Trait anxiety: r=.77 - .90  - Test-restest reliability State anxiety: r= .22 - .53  - Validity:  o Criterion validity: determined by correlation with other test scales (including FPI, EPI, EWL, BIV, Paranoid Depression Scale, Complaints List, and various subjective stress scales)  o Correlations of the Trait Anxiety Scale with the Lück and Timaeus Manifest Anxiety Measurement Scale (MAS) ranged from r = .73 to r = .90 | |

| **Falls Efficacy Scale-International Short (Short FES-I) ( Kempen, Yardley, van Haastregt, 2008)** | |
| --- | --- |
| Area | Declaration |
| fall-associated self-efficacy | - short FES-I, is a questionnaire with 7 items  - The person to be tested should indicate how great his or her reservations are about performing one of the questioned actions  - Selects between "no concerns at all"(1 point), "some" (2 points ), "quite a bit" (3 points), or "very much concerns" (4 points)  - sum of the points ranges between 7 (no fear of falling) and 28 points (maximum fear of falling) |
| **Quality criteria:**    - Internal consistency: Cronbachs α = .92  - Test-restest reliability: ICC= 0.83  - Validity:  o FES-I has an acceptable construct validity | |

Table 3 motor skills test procedure

| **Motor function** | | | |
| --- | --- | --- | --- |
| Test procedure | Motor area | declaration | |
| **Timed-Up-and-Go-Test**    **(D. Podsiadlo & S. Richardson, 1991)** | Mobility  restriction | | - The test person is to stand up from a sitting position without assistance, walk back and forth for 3 meters and sit down again (aids are allowed) - Score from ≤10s (Everyday mobility unrestricted) to ≥30 (Pronounced mobility restriction, usually intervention/ Need for assistive devices) |
|  | **Quality criteria:**  · Reliability: Interrater reliability: ICC = 0.91 (Rydwik,2011)  o Test-retest reliability: ICC = 0.96 - 0.99 (Flansbierr,2005)  · Validity:  o Criterion validity: Berg Balance Scale (r=0.81), Barthel Index (r=0.78) (Podsiadlo, 1991) | | |
| **FICSIT-4**    **(Rossiter-Fornoff et al. 1995)** | Static balance | | - The subject must perform four different stances with eyes open and closed (parallel, semi-tandem, tandem, and one-legged stance tests).      - Each stance is performed for a maximum of 10 seconds and then scored on a 5 point scale (0 points need help to keep from falling to 4 points able to stand 10 seconds safely). At the end, all the points obtained from the stands are added up to a total score. |
|  | **Quality criteria:**  · Reliability: Test-rests-reliability was good (r=.66) (Rossiter-Fornoff et al., 1995)  · Validity:  o Content validity was moderate (r=.20 - .52) (Rossiter-Fornoff et al., 1995) | | |
